# Draft Genome Sequence of *Bacillus* sp. Strain E25, a Biocontrol and Plant Growth-Promoting Bacterial Endophyte Isolated from Mexican Husk Tomato Roots (*Physalis ixocarpa* Brot. Ex Horm.)

**DOI:** 10.1128/MRA.01112-20

**Published:** 2021-01-07

**Authors:** Adrián Pérez-Equihua, Gustavo Santoyo

**Affiliations:** a Instituto de Investigaciones Químico Biológicas, Universidad Michoacana de San Nicolás de Hidalgo, Morelia, Michoacán, México; University of Arizona

## Abstract

*Bacillus* sp. strain E25 is an endophytic strain isolated from husk tomato roots in Michoacán, México, that displayed excellent biocontrol and plant growth-promoting activities under normal and salt stress conditions. This draft genome report confirms the presence of genes involved in direct and indirect mechanisms to stimulate plant growth and health.

## Abstract (precis)

*Bacillus* sp. strain E25 is an endophytic strain isolated from husk tomato roots in Michoacán, México, and displayed excellent biocontrol and plant growth-promoting activities under normal and salt stress conditions. This draft genome report confirms the presence of genes involved in direct and indirect mechanisms to stimulate plant growth and health.

## ANNOUNCEMENT

Plant growth-promoting bacteria (PGPB) have emerged as a wonderful alternative strategy to reduce or eliminate agrochemicals, commonly used to control pests and/or stimulate plant performance (1–3). One of the most common PGPB are members of the *Bacillus* genus. Bacilli are highly abundant in agricultural ecosystems; therefore, multiple strains have been isolated, characterized, and reported to influence crop growth and yield, including soil systems with problems of salinity (4).

Strain E25 was originally isolated as an endophyte of Mexican husk tomato roots (*Physalis ixocarpa* Brot. Ex Horm.) and maintained at −70°C in stock cultures containing nutrient broth (NB) and glycerol (40% [wt/vol]) (5). *Bacillus* sp. strain E25 exhibits multiple mechanisms of biocontrol and plant growth promotion, such as the production of indoleacetic acid, protease activity, and the emission of volatile organic compounds such as acetoin, 2,3-butanediol, and dimethyl disulfide, which under greenhouse conditions have been proven to stimulate the growth of tomato plants (5). Interestingly, strain E25 was able to modify its membrane components in order to protect its plant growth-promoting mechanisms from toxic effects of salt (6). Therefore, to elucidate the molecular mechanisms of biocontrol and plant growth promotion of strain E25, its genome was sequenced.

A single colony of the *Bacillus* sp. strain E25 was inoculated on 20 ml of NB and grown overnight at 28°C with agitation at 250 rpm. From the culture, total genomic DNA was extracted with a Wizard genomic DNA purification kit (Promega) according to the manufacturer’s instructions. The quality and quantity of the final DNA sample were evaluated via agarose-TAE gel (1.5%) electrophoresis and using a NanoDrop 1000 spectrophotometer (Thermo Scientific), respectively. DNA libraries were prepared using the Nextera DNA Flex library preparation kit (Illumina) according to the manufacturer’s user guide. Genomic samples were sequenced using the Illumina NovaSeq system (MR DNA, Lubbock, TX) with a coverage of 358× and a total of 18,999,694 reads. Read quality was assessed with FastQC analysis version 0.11.5 (7). Trimmomatic version 0.32 (8) was used to remove adapter sequences and low-quality bases. Reads were assembled *de novo* using SPAdes version 3.10.1 (9). Default parameters were used for all software. Multilocus sequence type, coding sequences, mRNAs, rRNAs, tRNAs, genes, and pseudogenes were annotated using the NCBI Prokaryotic Genome Annotation Pipeline (PGAP) (10).

The draft genome characteristics of strain E25 consist of a single 5,915,788-bp chromosome, with 34.7% G+C content, 6,130 total genes, 6,032 protein-coding genes, 5 noncoding RNAs (ncRNAs), 11 rRNA genes, 81 tRNA genes, and 294 pseudogenes. RAST genomic sequence analysis revealed the presence of potential genes involved in colonization of rhizospheric/root surface, such as those for chemotaxis (*che*), flagella and motility (*fli*, *mot*, and *flg*), iron acquisition (siderophores), or biofilm formation, among others. Interestingly, other genetic elements encoding transporter proteins, secretion and delivery systems, plant polymer degradation or modification, proteases, and type IV secretion systems were detected, which may be associated with an endophyte behavior (3, 11). *In silico* analysis of secondary metabolites using antiSMASH (12) showed that 17 gene clusters were relevant to producing active antagonistic compounds, including bacteriocins, siderophores, lanthipeptides, lipopeptides, ladderanes, and terpenes.

The genome sequence of *Bacillus* sp. strain E25 will contribute to unveiling molecular direct and indirect mechanisms for stimulating plant growth and health for further agro-biotechnological application.

### Data availability.

This full draft genome shotgun project has been deposited at GenBank under accession number CP031749.1. BioProject PRJNA476931, BioSample SAMN09461924, and SRA PRJNA476931 are also available.
